# Proton pump inhibitor-induced gut dysbiosis and immunomodulation: current knowledge and potential restoration by probiotics

**DOI:** 10.1007/s43440-023-00489-x

**Published:** 2023-05-04

**Authors:** Aneta Kiecka, Marian Szczepanik

**Affiliations:** Chair of Biomedical Sciences, Institute of Physiotherapy, Faculty of Health Sciences, Jagiellonian University Medical College, Kopernika 7a, 31-034 Kraków, Poland

**Keywords:** PPI, Proton pump inhibitors, Gut microbiota, Immunomodulation, Probiotics, SIBO, *Clostridioides difficile* infection, *Salmonella* infection

## Abstract

Proton pump inhibitors (PPIs) are the most commonly prescribed drugs for the treatment of non-erosive reflux disease (NERD), ulcers associated with non-steroidal anti-inflammatory drugs (NSAIDs), esophagitis, peptic ulcer disease (PUD), Zollinger–Ellison syndrome (ZES), gastroesophageal reflux disease (GERD), non-ulcer dyspepsia, and *Helicobacter pylori* eradication therapy. The drugs have the effect of inhibiting acid production in the stomach. According to research, PPIs can affect the composition of gut microbiota and modulate the immune response. Recently, there has been a problem with the over-prescription of such drugs. Although PPIs do not have many side effects, their long-term use can contribute to small intestinal bacterial overgrowth (SIBO) or *C. difficile* and other intestinal infections. Probiotic supplementation during PPIs therapy may provide some hope in the reduction of emerging therapy side effects. This review aims to present the most important effects of long-term PPI use and provides critical insights into the role of probiotic intervention in PPI therapy.

## Introduction

Proton pump inhibitors (PPIs) were introduced into medicine in 1989 and have become a safe way to treat disorders related to hydrochloric acid secretion ever since. As of 2015, there are six PPIs approved by the U.S. Food and Drug Administration (FDA) including omeprazole, ezomeprazole, lansoprazole, dexlanzoprazole, pantoprazole and rabeprazole [1]. PPIs are first-line drugs for the treatment of non-erosive reflux disease (NERD), ulcers associated with non-steroidal anti-inflammatory drugs (NSAIDs), esophagitis, peptic ulcer disease (PUD), Zollinger–Ellison syndrome (ZES), gastroesophageal reflux disease (GERD), and non-ulcer dyspepsia, among others [2, 3]. In addition, PPIs are used along with antibiotics in *Helicobacter pylori* eradication therapy [4]. PPIs show better inhibition of hydrochloric acid than histamine H2 receptor blockers [5]. Recently, there has been observed an over-prescription of PPIs among patients. Several studies unanimously found that among hospitalized patients, about 40–71.4% received therapy with PPIs during hospitalization. According to the researchers’ analysis, 65%-70%, of these patients had no real indication for acid-lowering drugs [6–8]. It appears that long-term use of PPIs can affect nutrient absorption, including calcium malabsorption [9]. Additionally, there are reports that bacterial translocation after PPIs use is potentially associated with the development of spontaneous bacterial peritonitis in patients with cirrhosis and ascites or cryptogenic hepatic abscess [10]. Long-term use of PPIs can also cause several side effects, such as iron deficiency anemia, vitamin B_12_ deficiency, and pneumonia [11, 12]. In addition, PPIs may interfere with bone metabolism, thereby increasing the incidence of fractures. Gastric juice antacids have been shown to inhibit phosphate absorption in the intestines. This process, in turn, can lead to hypophosphatemia and impaired bone mineralization [13]. Moreover, it is believed that in the case of aspiration pneumonia, inhibition of gastric acid production may contribute to colonization of the abdomen with organisms aspirated in the intensive care setting. It has been proven that patients on PPIs requiring ventilator assistance had a small but significant risk of pneumonia [14]. Studies show that PPIs can induce neurodegenerative diseases including dementia by affecting the brain-microbiota axis [15]. PPIs have been shown to increase amyloid protein deposition in the brain in a mouse model contributing to the induction of Alzheimer’s disease [16]. In addition, the risk of dementia increases by 1.4 times in PPI users [17]. Interestingly, it has also been shown that twice-daily PPI users are more likely to have COVID-19 infection, which is not observed in users of histamine-2 receptor antagonists [18].

It is proven that the gut microbiota plays a key role in metabolic, nutritional, physiological, defense, and immune processes in the human body, and its composition is closely related to emerging intestinal and extraintestinal diseases [19, 20]. Reports are showing that PPIs therapy can lead to changes in the composition of gut microbiota, which may be associated with adverse far-reaching effects of such therapy [21, 22].

It is important to find a method that attenuates the emerging side effects of PPIs administration. There are promising data showing how probiotics support PPIs therapy and regulate drug-induced intestinal dysbiosis [23].

This review aims to present the most important effects of long-term PPI use and provides critical insights into the role of probiotic intervention in PPI therapy.

## Effects of proton pump inhibitors

PPIs are benzimidazole derivatives, consisting of two heterocyclic groups that contain both pyridine and benzimidazole groups linked by a methylsulfinyl group [24]. Although each of the drugs classified as PPIs has different substitutions in their pyridine and/or benzimidazole rings, they show similar pharmacological properties [1]. PPIs are pro-drugs, effective only after protonation [25]. They are weak bases that are protected from premature activation and degradation by gastric acid through the use of gelatin capsules or as a powder to make a suspension [24]. Once moved from the stomach, PPIs are absorbed in the proximal part of the small intestine. The serum half-life is short, approximately 1–2 hours. After absorption, the circulation carries PPIs to the cells lining the abdomen, where they collect in acidic secretory tubules [1]. PPIs then undergo acid-catalyzed cleavage of the chiral sulfoxide bond to the active sulfenic acid and/or sulfonamide [24]. These compounds then bind covalently to cysteine residues in H^+^/K^+^ ATPase and have inhibitory effects on acid secretion until the replacement pumps are synthesized, i.e., for about 36 hours. The compounds form irreversible disulfide bonds with the H^+^/K^+^ -ATPase pump [26]. PPIs are used before meals as they require an active expression of H ^+^/K^+^ -ATPases in the tubules for binding to occur in response to a meal [1]. In the lumen of the stomach, the pH is maintained below 2. As has been shown for the treatment of diseases such as GERD, PUD, and *H pylori* infection, a gastric pH above 4 is recommended. When administered one tablet per day, PPIs can maintain a gastric pH above 4 for 10–16 hours [27, 28]. PPIs bind strongly to proteins and are degraded by hepatic cytochromes P450. Omeprazole and esomeprazole are metabolized by CYP2C19, while rabeprazole, lansoprazole and dexlanzoprazole are also metabolized by CYP2C19, but also show affinity for CYP3A4 [29]. After hepatic metabolism, the ultimate excretion of most benzimidazoles is through the kidneys, although lansoprazole and dexlanzoprazole are also excreted through the biliary tract [30].

## PPIs alter the gastrointestinal microbiota

**Fig. 1:**
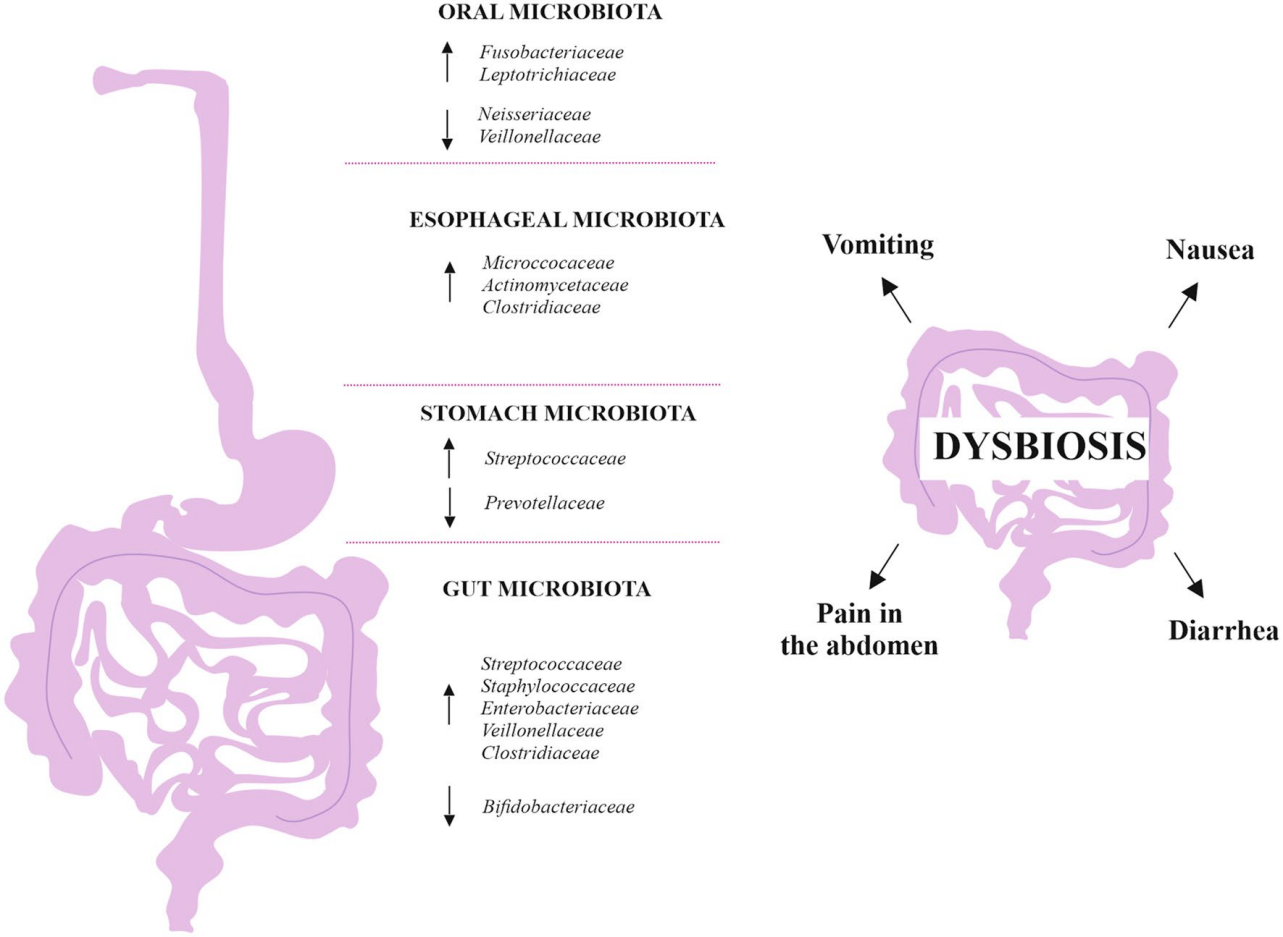
Alterations in the composition of the gut microbiota that occur in the oral cavity, the esophagus, the stomach, and the intestine after PPI treatment. (↑) indicates an increase in a specific bacterial family, while (↓) indicates a decrease in a specific bacterial family. *PPI* proton pump inhibitor

According to some studies, the use of PPIs may be associated with an increased risk of intestinal infections [31, 32]. Researchers hypothesize that PPIs alter the gut microbiota by directly affecting gastric acid (Fig. 1) [33, 34]. By reducing acidity in the stomach, PPIs allow more bacteria to overcome the barrier and enter the intestine. Imhann et al. conducted a study on 1,815 individuals from the Netherlands. The researchers collected fecal and oral samples to identify bacteria. They found that species such as *Rothia mucilaginosa, Rothia dentocariosa*, the bacterial genera *Scardovia* and *Actinomyces,* and the *Micrococcaceae* family were more numerous in the oral cavity of PPI users. Meanwhile, the esophageal microbiota showed increased amounts of *Micrococcaceae, Erysipelotrichaceae,* and *Enterobacteriaceae* [34]. Amir et al. showed that the use of PPIs can alter the esophageal microbiota, causing an increase in *Firmicutes* and a decrease in *Bacteroidetes* and *Proteobacteria* [35]. In a report by Bruno et al., the esophagus of PPIs users showed an increase in *Micrococcaceae*, *Actinomycetaceae*, *Clostridiaceae, and* a decrease in *Comamonadaceae,* while the stomach showed an increase in *Streptococcaceae* and a decrease in *Prevotellaceae* [36]. A study conducted by Mishiro et al. found that in healthy individuals, four weeks of esomeprazole administration resulted in an increase in *Fusobacterium* and *Leptotrichia* in the periodontal pocket, associated with a decrease in *Neisseria* and *Veillonella* in saliva and an increase in *Streptococcus* in fecal samples [37]. Jackson et al. studied fecal samples from 1827 patients. They showed that PPIs cause significantly lower bacterial counts in fecal samples and lower microbial diversity, as well as an increase in bacteria from the upper gastrointestinal tract, including an increase in *Streptococcaceae* [33]. Tsuda et al. obtained saliva, gastric fluid, and feces from 40 patients to assess the microbiota composition. The pH of the gastric fluid in PPIs users was >4, hence many oral bacteria could survive in the stomach. In the gastric fluid, the change in pH was associated with an increase in the beta diversity of microbiota. In addition, there was a reduction in the genus *Faecalibacterium* in the feces of subjects taking PPIs [38]. As is well known, this genus has anti-inflammatory properties. On the contrary, in a study conducted by Seto et al. after 28 days of PPIs in healthy volunteers*,* there was no change in the amount of *Faecalibacterium,* but there was a decrease in OTU in the fecal microbiota [39]. Hojo et al. administered PPIs, esomeprazole, to 20 patients with reflux esophagitis for 8 weeks. They then determined the composition of bacteria in feces and blood, in addition, the concentration of organic acids in feces and pH were determined. The study showed an increase in *Lactobacillus gasseri, Lactobacillus fermentum, Lactobacillus reuteri,* and *Lactobacillus ruminis* after treatment with PPIs [40]. *Lactobacilli* are considered to have beneficial effects on human health [41, 42], but there are several reports in which lactic acid bacilli caused severe infections such as bacteremia and liver abscesses in susceptible immunocompromised patients [43, 44]. However, Hojo et al. showed no differences in pH values and organic acid concentrations before and after PPIs [40]. A report by Sanduleanu et al. showed an increase in fecal and oropharyngeal bacteria in the gastric mucosa during the use of PPIs [45]. Shi et al. studied how the microbiota of gastric mucosa and feces changes in a group of patients with gastroesophageal reflux disease. They observed a higher abundance of *Streptococcaceae, Veillonellaceae, Acidaminococcaceae, Micrococcaceae,* and *Flavobacteriaceae* in the fecal microbiota of the PPI user group. Long-term use of PPIs was related to a lower relative abundance of *Pelobacter, Desulfuromonas, Alkanindiges, Koridiimonas, Marinobacterium,* and *Marinobacter*, and a higher relative abundance of *Luteimonas*, *Limonobacter, Herbaspirillum*, *Sphingobium*, *Phenylobacterium, Comamonas*, *Chryseobacterium*, *Duganella*, *Pedobacter*. However, short-term use of PPIs was associated with significantly lower levels of bacteria such as *Pelobacter*, *Desulfuromonas*, *Alcanlvorax*, *Kordiimonas*, *Desulfuromusa*, *Marinobacterium*, and *Marinobacter* [46]. Willems et al. conducted a case-control study on a group of 2239 hospitalized patients. This study showed that PPI users were 50% more likely to be infected with ESBL- or carbapenemase-producing *Enterobacterales*. This may be related to an increase in pH levels in the stomach and may contribute to the colonization of the intestines by pathogens [47]. In addition, several studies show that PPI use may be associated with the occurrence of microscopic colitis (MC), which is a disease of the colon accompanied by diarrhea. As is already known, MC is associated with gut microbiota dysbiosis. Individuals with MC show lower bacterial diversity and *Prevotella* enrichment in stool samples [48]. Among patients with cirrhosis, PPIs may be a risk factor for hepatic encephalopathy. This occurs through the translocation of intestinal bacteria. Moreover, this translocation may lead to spontaneous bacterial peritonitis [49].

The conducted studies are interesting, but they are mainly based on the evaluation of the microbiota of saliva, gastric juice, or feces. Therefore, they do not necessarily reflect events occurring in the small intestine (duodenum, cecum, and ileum). For example, the pH environment of the duodenum is different from that of the ileum or the colon, which may affect colonization by particular bacteria and influence the immune response [46].

## PPIs modulate the immune response

PPIs are deposited in the stomach, where the nitrogen atoms of pyridine and benzimidazole are protonated, converting the prodrug into active tetracyclic sulfenamides, which bind and inhibit proton pumps [50]. PPIs block p-type H^+^/K^+^ ATPases [51]. Also, neutrophils and endothelial cells have vacuolar H^+^ ATPases. They can pump acid into the extracellular space and lysosomes [52, 53]. Upon activation of neutrophils, vacuolar H^+^ ATPases pump H^+^ into the phagolysosome [54, 55]. Acidification of lysosomes occurs, which mediates the oxidative burst of neutrophils and leads to the release of toxic reactive oxygen species (ROS) [51]. Wandall et al. in their study showed that omeprazole *in vitro* inhibits formyl-methionyl-leucyl-phenylalanine (fMLP)-stimulated chemotaxis and neutrophil superoxide production [54]. Whether the effect of PPIs on neutrophils is due to the inhibition of v-type H^+^ ATPases is not fully explored [51].

It is important to note that most of the studies conducted on the effects of PPIs on the immune response were performed *in vitro* using cell lines. Thus, the data may not fully reflect the changes that occur in a living organism.

### Cytokines

Tanigawa et al. investigated the effect of lansoprazole on the production of tumor necrosis factor-α (TNF-α) and interleukin-1β (IL-1β) induced by lipopolysaccharide (LPS) and extract *H. pylori* (HpWE). Lansoprazole (100 μM) significantly reduced the mRNA expression and production of TNF-α and IL-1β by the human monocyte cell line (THP-1) stimulated with LPS and HpWE. This report indicated that lansoprazole inhibited the phosphorylation and degradation of inhibitory factor κB-α (IκB-α) and phosphorylation of extracellular signal-regulated kinase (ERK) induced by LPS and HpWE in THP-1 cells [56, 57]. Ubagai et al. investigated the effect of lansoprazole on gene expression, especially immunomodulatory genes, in human polymorphonuclear leukocytes (PMNs) activated by LPS. Lansoprazole (0-10 mg/ml) was added to PMNs that were stimulated with LPS (100 ng/ml). It was shown that mRNA expression levels of CXCR1/2 and TNF-α were suppressed in a dose-dependent manner. CD14 gene expression levels were also reduced by lansoprazole. This was one of the pieces of evidence that lansoprazole can inhibit the biological functions of PMNs, such as chemotaxis and the production of pro-inflammatory cytokines [58]. In addition, Cortes et al. showed that treatment of peripheral blood mononuclear cells (PBMCs) as well as bone marrow cells (HL-60), lymphoid cells (HUT-78, M.12), fibroblasts (NIH3T3), lung cells (A549) and colon cells (HT-29) with omeprazole inhibited STAT6 phosphorylation induced by interleukin-4 (IL-4) and interleukin-13 (IL-13) in a dose-dependent manner. Treatment of cells with omeprazole also inhibited the activation of Janus kinase-1 (JAK-1) and Janus kinase-3 (JAK-3) by IL-4 [59]. Handa et al. stimulated gastric epithelial cells (MKN45) and human umbilical vein endothelial cells (HUVEC) with an aqueous extract of *H. pylori* and IL-1β, then applied PPIs to evaluate their effects on cytokine production. They also evaluated the effects of PPIs on interleukin-8 (IL-8)-induced PMN transendothelial migration and on the change in cytoplasmic calcium concentration in PMN stimulated with formyl-methionyl-leucyl-phenylalanine (fMLP). The researchers observed that *H. pylori* and IL-1β induced an increase in IL-8 production by MKN45 and HUVEC cells, and NF-κB activation, which was significantly inhibited by PPIs administration. PPIs also inhibited IL-8-induced PMN transendothelial migration and fMLP-induced increase in PMN cytosolic calcium [60]. It was also shown that in cultured human tracheal cells, PPIs reduced the levels of various pro-inflammatory cytokines, including IL-6, IL-8, and TNF-α [61]. Nakatake et al. analyzed the effects of lansoprazole on inducible nitric oxide synthase (iNOS) induction and nitric oxide (NO) production as well as related signaling pathways *in vitro* on primary cultured rat hepatocytes and *in vivo* using a rat model of liver injury. The team found that lansoprazole inhibited iNOS induction and NO production *in vitro*, in part by inhibiting NF-κB activation. In addition, lansoprazole also decreased TNF-α and CXCL-1 mRNA levels, which shows that lansoprazole inhibits hepatic mRNA expression of iNOS, TNF-α, CXCL-1, IL-1β, and IL-6 in treated rats, and thus, prevents the expression of these pro-inflammatory mediators at the transcriptional level [62]. Ghebremariam et al. studying idiopathic pulmonary fibrosis (IPF) using cell cultures showed that esomeprazole inhibited the expression of pro-inflammatory molecules, including vascular cell adhesion molecule-1, iNOS, TNF-α, IL-1β and IL-6 [63].

### Natural killer cells

It has been shown that PPIs, in addition to their effects on neutrophils and cytokines, can also modulate the activity of natural killer cells (NK cells). Studies show that PPIs including omeprazole and lansoprazole reduce the cytotoxic activity of NK cells in a dose-dependent manner [64, 65]. Lysosomotropic factors are observed to significantly reduce NK cell cytotoxicity *in vitro* [66]*.* This shows that the vacuole system plays a significant role in the process of NK cell cytotoxicity. Therefore, it can be concluded that omeprazole can reduce NK cell cytotoxicity by concentrating on acidic organelles such as lysosomes. However, the effect on NK cells may also take place through other pathways. Alkim et al. showed that taking omeprazole for four weeks at normal therapeutic doses (20 mg) significantly reduces NK cell function. Omeprazole significantly inhibits both the conjugation and cytotoxicity of mononuclear cells exerted on K562 cells, which are targets of NK cells [67]. However, it is difficult to explain whether their finding was related to omeprazole’s tendency to concentrate in acidic environments. In addition, the authors suggested that there may be an interaction between omeprazole and one of the membrane structures in the vacuole system (e.g., V-ATPase), which can disrupt membrane recycling and alter plasma membrane fluidity. Disruption of cell membrane function can lead to inhibition of conjugation formation between NK cells and K562 target cells [67, 68].

## Intestinal-related side effects of PPI use

Increasingly, physicians are prescribing PPIs for long-term, sometimes lifelong use, often in the lack of proper indications. Hence, there is a growing concern about the potential side effects of such long-term therapy. One of the body’s main non-specific defense mechanisms and an important barrier to pathogens is the inactivation of ingested microorganisms by gastric acid. Hypochlorhydria and achlorhydria are associated with an increased risk of intestinal infections [69, 70]. Researchers have shown increased concentrations of bacteria in the stomach of healthy individuals taking omeprazole daily for two weeks [71, 72]. It can be seen that this increase in bacterial concentration was reduced after omeprazole was discontinued [71]. Intestinal side effects of PPIs therapy can include small intestinal bacterial overgrowth (SIBO) and intestinal infections caused by *C. difficile* or *Salmonella* spp*.* among others, as well as other pathogens [73–75].

### Small intestinal bacterial overgrowth

Long-term treatment with PPIs affects the microbiota of the small intestine, through which SIBO can occur, due to the loss of the “defense barrier” that is gastric acid [73]. SIBO is a condition in which the amount of bacteria in the intestine is more than 10^5^ per ml of upper intestinal aspirate. While the normal value will be less than 10^4^ per ml of upper intestinal aspirate [76, 77]. SIBO is a syndrome characterized by increased and/or abnormal types of bacteria in the small intestine. It can be asymptomatic or resemble irritable bowel syndrome with nonspecific symptoms including bloating, abdominal discomfort, abdominal pain, or diarrhea. More severe cases develop weight loss, fat stools, malnutrition, hepatic changes, rosacea, joint pain and anemia, tetany in hypocalcemia caused by vitamin D_3_ deficiency, metabolic bone disease, and polyneuropathy caused by vitamin B_12_ deficiency. Several mechanisms prevent bacterial overgrowth in the intestine including gastric acid secretion, intestinal motility, intact ileocecal valve, and bacteriostatic properties of pancreatic and biliary secretion [78]. Some reports indicate that the use of PPIs affects the occurrence of SIBO [79]. As early as 1994, there were the first attempts to evaluate whether the use of PPIs affects SIBO. Nelis et al. administered omeprazole 20 mg per day to patients for a period of 4–26 weeks. After testing for bacterial overgrowth in the intestine, they concluded that there was no relationship between PPIs use and SIBO [80]. Similar tests were conducted by Pereira et al., in 1998, who administered omeprazole 20 mg per day to a group of elderly patients. They took duodenal aspirates from the patients before and after the test. Before the test, patients had bacterial counts <10^4^ colony-forming units (CFU)/mL (96% of patients), while after PPIs therapy, 43% of patients developed bacterial counts > 10^5^ CFU/mL [81]. Lombardo et al. conducted a glucose-hydrogen breath test (GHBT) for SIBO in 200 patients with gastroesophageal reflux disease who used PPIs for 36 months and non-PPIs users with irritable bowel syndrome (IBS) and healthy controls. SIBO was found in 50% of PPI users, 24.5% of IBS patients, and 6% of healthy controls. The conclusion was drawn that long-term use of PPIs affects the occurrence of SIBO [82]. Compare et al. treated patients with erosive reflux disease and gastroesophageal reflux disease with esomeprazole 20 mg twice daily for 6 months. The patients underwent a GHBT test before treatment and after 8 weeks of therapy. It was revealed that after 8 weeks of PPIs therapy, patients complained of bloating (43%), flatulence (17%), abdominal pain (7%), and diarrhea (2%). After 6 months of therapy, the incidence of intestinal symptoms continued to increase [83]. A 2018 meta-analysis by Su et al. showed that studies using small intestinal aspirate culture I GHBT have observed an association between PPI use and the occurrence of SIBO [84]. Although data on the relationship between PPIs use and SIBO incidence are inconclusive, Weitsman et al. in their 2022 report showed that PPIs users have an altered gut microbiota, but this is not related to an increased risk of SIBO [85]. What is more interesting is that more recent reports indicate a potential link between intestinal microbial composition and SIBO. In patients diagnosed with SIBO, the composition of gut microbiota shows significantly reduced α diversity compared to patients without SIBO. In addition, there is an increased relative abundance of *Streptococcus* and a decreased relative abundance of *Bacteroides* compared to patients without SIBO [86]. In 2019, Shin et al. collected aspirate and small intestinal mucosa samples from patients with SIBO. They showed reduced α diversity, but no differences in β diversity in patients with SIBO compared to patients without SIBO. They showed no differences in the abundance of individual bacterial taxa in aspirates [87]. There are reports that SCFAs are involved in the pathogenesis of SIBO, affecting intestinal motility [88]. Moreover, Rizos et al. showed that SIBO is associated with inflammation. Elevated levels of pro-inflammatory cytokines were found in patients with SIBO: IL-1β, IL-6, and TNF-α in duodenal fluid [89].

However, the available data on the relationship between dysbiosis and SIBO are incoclusive. The occurrence of dysbiosis does not necessarily result in disease symptoms. In addition, studies are showing that intestinal dysbiosis is not associated with SIBO. For example, patients with cirrhosis often have SIBO, but it is not associated with intestinal dysbiosis [90]. Hence, further research is needed to determine whether there is a relationship between altered intestinal microbiota composition and SIBO.

### Enteral infections

*C. difficile* infection is among the most common nosocomial infections [91]. The course of the disease varies, in some people, it is an asymptomatic carrier, and in others, it can lead to severe diarrhea, colonic dilatation, and even death [92]. The pathophysiology of *C*. *difficile* infection is not yet understood. Two toxins, TcdA and TcdB, encoded by tcdA and tcdB, respectively, are suggested to cause infection. Data show that these toxins are responsible for the clinical symptoms of *C. difficile* infection by causing damage to intestinal epithelial cells [93]. *In vitro* exposure to these toxins, induces the release of pro-inflammatory cytokines [94, 95]. It is suspected that intestinal dysbiosis and induced inflammation may be responsible for *C. difficile* infection [95, 96]. Inhibition of gastric acid production may contribute to an increased tendency for intestinal infections. Gastric acid is an important barrier to many pathogenic microorganisms [97, 98]. Aseeri et al. studied the relationship between the occurrence of diarrhea related to *C. difficile* infection and the use of PPIs. They confirmed that there was a higher risk of diarrhea related to *C. difficile* infection in patients using PPIs [74]. Dial et al. showed that the incidence of *C. difficile* infection was 4.4% in patients not taking PPIs, compared to 9.3% in patients taking PPIs, showing a statistically significant relative risk ratio of 2.1 [99]. Another report found that the incidence of *C. difficile* infections increased from nearly 37% to about 63% among patients taking PPIs [100]. Janarthanan et al. conducted a meta-analysis in which they analyzed the results of 23 studies involving nearly 300,000 patients. They showed that 65% of patients developed diarrhea associated with intestinal colonization by *C. difficile* after the use of PPIs [101]. In a mouse model of *C. difficile-*induced colitis, it was shown that administration of PPIs led to changes in fecal consistency and weight loss. In addition, colon samples showed more neutrophilic infiltration, epithelial damage, and production of pro-inflammatory cytokines in PPIs-treated mice. Mice with colitis treated with PPIs showed an increase in the expression of pro-inflammatory cytokines such as IL-1β, IL-6, interleukin-17A (IL-17A), TNF-α, interferon-γ ( IFN-γ), macrophage inflammatory protein-2 (MIP-2), and monocyte chemotactic protein-1 (MCP-1) [102]. PPIs have been shown to cause a decrease in bacteria of the *Ruminococcocaeae* family and the genus *Bifidobacterium* and an increase in bacteria of the *Gammaproteobacteria* class, the *Enterococcaceae*, *Enterobacteriaceae,* and *Lactobacillaceae* families and the *Enterococcus and Veillonella* genera, which are associated with increased susceptibility to *C. difficile* infection [103, 104]. Thus, PPIs may increase not only the risk of *C. difficile* infection but also the associated mortality [105].

Salmonellosis is a gastrointestinal infection caused mainly by non-double serotypes of *Salmonella* spp. [106]. Wu et al. showed that Nontyphoid salmonellosis patients more often include PPI users [107]. A case-control study conducted in 2006 showed that one of the causes of *Salmonella* spp. infection is the recent use of PPIs [75]. The reason may be the neutralization of gastric acid by PPIs, thus the antisecretory effect of PPIs may inhibit the protective antimicrobial effect of gastric juice and thus may promote *Salmonella* spp*.* infection [108, 109]. A report by Lee et al. showed that treatment with PPIs reduced butyrate levels in various parts of the small and large intestines [110]. As shown in studies on bacterial strains, butyrate-produced SCFAs significantly affect the inhibition of *Salmonella* spp. infection [111]. An increase in IL-1β and TNF-α expression is also observed during *Salmonella spp*. infection [112]. Previous reports show that H2 blocker drugs that inhibit the secretion of hydrochloric acid by gastric lining cells (cimetidine) used after surgery caused a decrease in IL-8, neutrophil elastase, and C-reactive protein as well as a concomitant increase in lymphocyte regeneration rate, showing the immunomodulatory properties of acid-regulating compounds [113]. As already known, neutrophil elastase alters the gut microbiota, causing increased colonization of *Salmonella* spp. [114].

## Is it worth using probiotics during PPIs therapy?

Probiotics are live microorganisms that, when administered in adequate amounts, provide health benefits to the host [115]. As already described, long-term use of PPIs can lead to intestinal dysbiosis, thereby increasing susceptibility to infection resulting in intestinal disease. Recently, it has been suggested that probiotic supplementation should additionally be included during PPIs therapy to increase its effect. In addition to increasing the effect of PPIs therapy, probiotic supplementation can potentially inhibit intestinal dysbiosis and the side effects of long-term PPIs use. Belei et al., in their project, for 12 weeks of PPIs treatment in children with gastroesophageal reflux disease, additionally administered the probiotic *Lactobacillus reuteri* DSM 17938 to the study group. After the treatment period, it was shown that intestinal dysbiosis occurred in 56.2% of the children in a group that was not given the probiotic, while in a group that received the probiotic, dysbiosis occurred in only 6.2% of the children [116]. There are several potential mechanisms through which the health-promoting activity of probiotics in PPIs therapy may occur. First of all, it is noted that PPIs lead to intestinal dysbiosis. One mechanism of action of probiotics may be competitive exclusion. This refers to when one bacterial species competes for receptor sites in the gastrointestinal tract more than others. The pathways by which probiotics compete for receptor sites are largely unknown [117]. Probiotics act as a “barrier” to limit the proliferation of pathogenic bacteria [23]. Studies show that certain probiotic metabolites may play a role in modulating various metabolic and signaling pathways in cells [118]. In addition, lactic acid bacilli and bifidobacteria can produce bacteriocins that inhibit the proliferation of certain pathogens [119]. Probiotics show enzymatic activity and interact with bile acids in the intestinal lumen, modifying bile acid metabolism [120]. Additionally, probiotics modulate the host immune response [121]. More than 70% of immune cells are located in the intestines, especially in the small intestine, forming gut-associated lymphoid tissue (GALT). The GALT contains structures such as Peyer’s patches, lymph nodules, lymphocytes, and mucous membranes [122]. Probiotic bacteria have been shown to induce a pattern of dendritic cell (DC) maturation characterized by the release of small amounts of TNF-α and Interleukin 12 (IL-12), with increased levels of IL-10, that inhibit the production of pro-inflammatory Th1 lymphocytes [123]. Reports show that probiotics have activity against SIBO, *C. difficile* infection, *Salmonella* spp. infection, i.e., infections that constitute side effects of PPIs therapy [124, 125].

However, in addition to the beneficial effects of probiotics, there are reports that their long-term administration can cause fungemia and bacteremia in some cases [115]. Hence, before they would be routinely administered in PPI therapy, it would be necessary to extensively study their health-promoting properties and effects on the microbiota in patients treated with PPIs.

### Small intestinal bacterial overgrowth

There are reports that probiotics, for example, the probiotic strain *Lactobacillus casei* effectively modulates the small intestinal microbiota, which is associated with a reduction in SIBO [125]. Piano et al. in their project administered four selected probiotics *Lactobacillus rhamnosus* LR06 (DSM 21981), *Lactobacillus pentosus* LPS01 (DSM 21980), *Lactobacillus plantarum* LP01 (LMG P-21021) and *Lactobacillus delbrueckii* LDD01 (DSM 22106) to patients using PPIs for more than 12 months. Administration of the probiotic mixture significantly reduced bacterial overgrowth in patients treated with long-term PPIs [126]. In contrast, Hegar et al. conducted a study on 70 children who were orally administered omeprazole 20 mg per day for 4 weeks. 36 children were additionally given the probiotic *Lactobacillus rhamnosus* R0011 (1.9 × 109 CFU) and *Lactobacillus acidophilus* R0052 (0.1 × 109 CFU) during PPIs therapy, while 34 received a placebo. After one month of treatment, it was found that the administered probiotic did not reduce the risk of developing SIBO [127]. Belei et al. showed in a group of 128 children with GERD treated with PPIs that administration of an additional probiotic strain of *Lactobacillus reuteri* DSM 17938 reduced the incidence of intestinal dysbiosis and inhibited the risk of SIBO [116]. Kwak et al. administered *Bifidobacterium bifidum, Bifidobacterium lactis, Bifidobacterium longum, Lactobacillus acidophilus, Lactobacillus rhamnosus,* and *Streptococcus thermophilus* to 53 patients with chronic hepatic disease. After 4 weeks, changes in fecal bacterial composition, SIBO, and intestinal permeability were investigated. Short-term use of probiotics was found to alleviate SIBO [128]. A pilot study by Khalighi et al. showed that the administration of a probiotic for SIBO therapy alleviates side effects of PPI treatment [129]. It has been shown that in patients who have undergone Roux-en-Y gastric bypass surgery, after which bacterial overgrowth in the intestine and impaired intestinal motility can occur, administration of probiotics of the *Lactobacillus* species can inhibit the onset of SIBO [130]. In contrast, other researchers have shown that *L. acidophilus* and *B. lactis* are effective in reducing bloating, but do not affect SIBO [131]. In Crohn’s disease, probiotics have not been shown to be effective in disease remission [132].

### Enteral infections

There are reports showing that probiotics may be effective in attenuating *C. difficile* infection. Riperta et al. studied *Bacillus clausii* to neutralize the toxin produced by *C. difficile*, the main virulence factor of this pathogen. Incubation of the supernatant containing *C. difficile* toxin with *B. clausii* protected the cell line from the toxin’s cytotoxicity [133]. This was associated with the production of serine protease by *B. clausii* [134]. In a study conducted by Nagamine et al., the addition of probiotics such as *Bacillus mesentericus* and *Clostridioides butyricum* to antibiotic therapy in elderly patients undergoing orthopedic surgery inhibited the eventual emergence of *C. difficile* infection [135]. Gao et al. administered the probiotics *Lactobacillus acidophilus* CL1285 and *Lactobacillus casei* LBC80R Bio-K^+^ CL1285 to hospitalized patients who could develop *C. difficile* infection due to antibiotic therapy. They showed that this probiotic mixture could inhibit eventual *C. difficile* infection [136]. Inhibition of *C. difficile* has also been shown following the use of probiotics such as *Bifidobacterium bifidum,* and *Saccharomyces boulardii* [137, 138]. Studies show that the beneficial health-promoting effects of probiotics may be largely related to their effects on the gut microbiota. SCFA concentration is important for *C. difficile* infection, according to the study. At low SCFA concentrations, greater susceptibility to *C. difficile* has been found [139–141]. It has been shown that higher SCFA concentrations are important to prevent diarrhea [142]. Reduced SCFA is associated with intestinal leakiness, which can lead to intestinal dysbiosis and inflammation [129]. Studies show that probiotic strains have the ability to stimulate SCFA production by affecting the modulation of the gut microbiota [143–145].

The effect of probiotics on *Salmonella* spp. infection has been reported. Zihler et al. showed the inhibitory effect of *Bifidobacterium thermophilum* RBL67 on *Salmonella spp.* in vitro culture [146]. Among others, *Bifidobacterium infantis* and *Bifidobacterium breve* from the VSL#3 probiotic cocktail were shown to improve T84 cell epithelial integrity and resistance to *Salmonella* spp. invasion [147]. It has been suggested that probiotics use different mechanisms to favorably modulate the intestinal epithelium and mediate protection against *Salmonella* spp*.* [148]. In 2012, Bermudez-Brito et al. showed that incubation of human DCs with *Salmonella spp.* and live *L. paracasei* bacteria significantly reduced the ability of *Salmonella* spp. to induce IL-6, IL-8, IL-12p 70, and TNF-α. In 2013, the same team showed that the probiotic *Bifidobacterium breve* CNCM I-4035 has immunomodulatory effects on human intestinal dendritic cells and thus counteract *Salmonella enterica serovar Typhi* infection*.* In this study, *Bifidobacterium breve* CNCM I-4035 reduced pro-inflammatory cytokines and chemokines in human intestinal DCs provoked with *S. typhi* [149].

## Conclusion

Proton pump inhibitors including omeprazole, ezomeprazole, lansoprazole, dexlansoprazole, pantoprazole, and rabeprazole are first-line drugs for the treatment of conditions such as NERD, NSAID-related ulcers, PUD, ZES, GERD, and others. In addition, they are used during antibiotic therapy for *H. pylori* eradication therapy. PPIs inhibit gastric acid secretion by changing pH and acting on H^+/^K^+^ ATPases. Recently, much attention has been paid to the effect of various substances on the gut microbiota. It turns out that the microbiota may be involved in the pathogenesis of many diseases. The gut microbiota can modulate lipid accumulation, lipopolysaccharide content, and the production of short-chain fatty acids, which affect food intake, inflammation, or insulin signaling. It is believed that PPIs can alter gut microbiota by affecting gastric acid production. An increase in beta diversity can be observed in PPI users. In addition, there is a reduction in the genus *Faecalibacterium*, which is known to have anti-inflammatory properties. Moreover, some studies show an increase in *L. gasseri, L. fermentum, L reuteri, L. ruminis*. The fecal microbiota of PPI users shows a higher abundance of *Streptococcaceae, Veillonellaceae, Acidaminococcaceae, Micrococcaceae,* and *Flavobacteriaceae*. PPIs also have immunomodulatory properties. This may be due to the fact that neutrophils and endothelial cells possess vacuolar (v-type) H^+^ ATPases, and these may be susceptible to the effects of PPIs. However, it is not clear whether the effect on neutrophils is due to this property alone. Data show that lansoprazole, among others, is related to decreased mRNA expression as well as inhibition of TNF-α and IL-1β production. In addition, there are reports in which PPIs including omeprazole and lansoprazole reduce the cytotoxic activity of NK cells.

The problem becomes that PPIs are often prescribed to patients who have no clear indication for their use. Due to the potentially low number of side effects, these drugs are prescribed too often, sometimes even for long-term or lifetime use. However, it turns out that long-term use of PPIs can contribute to side effects including SIBO, or intestinal infections. The challenge is to understand the exact causes of this relationship. Increasingly, intestinal dysbiosis, a consequence of PPI use, is being cited as a cause of side effects. An underestimated element in the pathogenesis of SIBO, *C. difficile,* and *Salmonella* spp. infections may be the intestinal microbiota and immune response. For example, it appears that individuals diagnosed with SIBO show significantly altered alpha diversity in the composition of gut microbiota compared to healthy individuals. In addition, there is an increased abundance of *Streptococcus* and a decreased abundance of *Bacterioides*. Furthermore, there are reports that SCFAs are involved in the pathogenesis of SIBO, affecting intestinal motility and that individuals with SIBO have an increase in IL-1β, IL-6, and TNF-α in the duodenal fluid. However, it is not possible to determine whether there is a relationship between SIBO and intestinal dysbiosis. As mentioned, there are many studies in which this relationship has not been proven. Extensive research in this area would be needed. A different side effect probably also related to intestinal dysbiosis after PPIs is the occurrence of *C. difficile* infection. It is claimed that gastric acid inhibition can contribute to the disruption of barriers and the entry of bacteria into the intestines where dysbiosis can occur. Data are showing that the use of PPIs leads to a decrease in *Ruminococcaceae* bacteria and the genus *Bifidobacterium* as well as an increase in *Gammaproteobacteria Enterococcaceae* and *Enterobacteriaceae* and *Lactobacillaceae* and the genera *Enterococcus* and *Veillonella*, which have already been shown to be associated with susceptibility to *C. difficile*. The report shows that treatment with PPIs reduces butyrate and thus SCFA levels, which can also lead to *Salmonella* spp. infection.

There has been an ongoing search for a way to mitigate the side effects of long-term PPIs use including the inclusion of probiotics, among others, to alleviate the dysbiotic changes created in the intestine after PPIs therapy. Probiotic strains such as *Lactobacillus reuteri* DSM 17938 have appeared promising, showing mitigating effects in children on PPIs therapy. Other interesting strains with potential protective function include *L. rhamnosus* LR06 (DSM 21021) or *L. pentosus* LPS01 (DSM 21980), these probiotic mixtures reduce intestinal battery overgrowth, potentially inhibiting SIBO induced by PPIs therapy. In addition, it can be observed that probiotics can inhibit PPIs-induced intestinal dysbiosis, and can affect the production of SCFAs, which are inhibited after PPIs therapy, and are as seen important in the pathogenesis of SIBO, or *C. difficile* infection.

The problem in evaluating the effects of PPIs on the gut microbiota and immune response is that there are not enough studies comparing long-term and short-term use of PPIs and their effects on immune cells and the microbiota. Unfortunately, often advances in the treatment of underlying diseases provide a reason to abandon mild side effects. Unfortunately, these initially mild side effects after the passage of time and continued therapy as described above sometimes for life can trigger SIBO or *C. difficile* infection, among others, which is known to be fatal in 2-6% of cases. Future research should focus on the evaluation of the effects of PPIs on the immune response, including cell function, and cytokine production. It is important to expand the knowledge in this area, as the studies described in this review were conducted using *in vitro* methods on cell lines, which may not reflect what happens in a living organism. It would also be valuable to understand in detail the mechanisms by which PPIs can modulate the immune response. In addition, it would be necessary to assess what the difference in the immune response is between long-term and short-term use of PPIs. It is known that PPIs alter the composition of gut microbiota, but it would be valuable to know whether this occurs only by disrupting the protective barrier of gastric acid, or whether other mechanisms contribute to this. Furthermore, referring to reports evaluating the attenuation of the side effects of long-term PPIs after probiotic therapy, it would be useful to assess what differences there are in gut microbiota composition and immune response after PPIs plus probiotic combination therapy. It should be noted, however, that most of the studies described here addressed the composition of the microbiota in saliva or fecal samples, which does not necessarily correspond to the microbiota composition in specific regions of the intestine in a living organism.

Our unpublished data suggest that PPI-induced dysbiosis inhibits contact hypersensitivity (CHS) reaction in mice which is an animal model of allergic contact dermatitis (ACD) in humans. ACD is a classical T cell-mediated disease, classified as type IV hypersensitivity reaction. Interestingly, the immune response observed in type IV hypersensitivity is also involved in the numerous T cell-mediated autoimmune diseases such as multiple sclerosis, Crohn’s disease, and rheumatoid arthritis. Thus, studies unraveling the influence of PPI-induced dysbiosis on CHS could shed light on immune responses observed in various autoimmune disorders.

The limitation of this review and at the same time the challenge of future years becomes the fact that research on probiotic use in PPI therapy is at a very early stage. This issue has huge clinical implications, and more direct evidence of their efficacy will be needed before the floodgates for probiotics can be opened. Unfortunately, sampling the gut microbiota is a challenge as duodenal aspiration does not reflect the consequences of specific diets, supplements, and medications. In addition, it cannot show the postprandial release of biliary and pancreatic enzymes, which have their effects on the intestinal microbiota composition. In the same way, stool samples are predictably different from small intestine samples and may not be sufficient to determine relationships between gut microbial composition and the use of probiotic therapy. Similarly, data on changes in intestinal permeability to altered intestinal microbiota and immune response are inconclusive and hypothetical, thus requiring further studies.

In summary, PPIs change the composition of gut microbiota by, among others, altering pH and affecting the modulation of the immune response. In addition, many studies show that long-term use of PPIs may be associated with serious side effects, including SIBO and intestinal infections including *Salmonella* spp. and *C. difficile*. However, PPI therapy at this point is the only treatment with the least side effects for many diseases including NERD, NSAIDs, PUD, ZES, and GERD. Indications are that probiotics may have an effect on inhibiting intestinal dysbiosis after PPIs and may help alleviate the side effects of PPIs therapy. However, further studies related to their efficacy in patients using PPIs are needed before they could be incorporated into therapy.

## Data Availability

Not applicable.

## Abbreviations

CFU: Colony forming units
ERK: Extracellular signal-regulated kinases
Fmlp: N-Formylmethionine-leucyl-phenylalanine
GALT: Gut-associated lymphoid tissue
GERD: Gastroesophageal reflux disease
GHBT: Glucose-hydrogen breath test
IBS: irritable bowel syndrome
IFN-γ: Interferon-γ
IL-1β: Interleukin - 1 β
IL-4: Interleukin - 4
IL-6: Interleukin - 6
IL-8: Interleukin - 8
IL-10: Interleukin -10
IL-12: Interleukin -12
IL-17A: Interleukin - 17A
IEL: intraepithelial lymphocytes
iNOS: Nitric oxide synthase
IPF: idiopathic pulmonary fibrosis CFU
JAK-1: Janus kinase-1
JAK-3: Janus kinase-3
LPS: Lipopolysaccharide
MCP-1: Monocyte chemotactic protein-1
MIP-2: Macrophage inflammatory protein-2
NADPH: Nicotinamide adenine dinucleotide
NERD: Non-erosive reflux disease
NO: Nitric oxide
NK cells: Natural killer cells
PMN: Polymorphonuclear leukocyte
PPIs: Proton pump inhibitors
PUD: Peptic ulcer disease
ROS: Reactive oxygen species
SCFA: Short chain fatty acid
SIBO: Small intestine bacterial overgrowth
TNF-α: Tumor necrosis factor-α
ZES: Zollinger–Ellison syndrome
